# Mechanistic Model of Natural Killer Cell Proliferative Response to IL-15 Receptor Stimulation

**DOI:** 10.1371/journal.pcbi.1003222

**Published:** 2013-09-12

**Authors:** Yun M. Zhao, Anthony R. French

**Affiliations:** 1 Division of Pediatric Rheumatology, Department of Pediatrics, Washington University School of Medicine, St Louis, Missouri, United States of America; 2 Department of Biomedical Engineering, Washington University, St Louis, Missouri, United States of America; Memorial Sloan-Kettering Cancer Center, United States of America

## Abstract

Natural killer (NK) cells are innate lymphocytes that provide early host defense against intracellular pathogens, such as viruses. Although NK cell development, homeostasis, and proliferation are regulated by IL-15, the influence of IL-15 receptor (IL-15R)-mediated signaling at the cellular level has not been quantitatively characterized. We developed a mathematical model to analyze the kinetic interactions that control the formation and localization of IL-15/IL-15R complexes. Our computational results demonstrated that IL-15/IL-15R complexes on the cell surface were a key determinant of the magnitude of the IL-15 proliferative signal and that IL-15R occupancy functioned as an effective surrogate measure of receptor signaling. Ligand binding and receptor internalization modulated IL-15R occupancy. Our work supports the hypothesis that the total number and duration of IL-15/IL-15R complexes on the cell surface crosses a quantitative threshold prior to the initiation of NK cell division. Furthermore, our model predicted that the upregulation of IL-15Rα on NK cells substantially increased IL-15R complex formation and accelerated the expansion of dividing NK cells with the greatest impact at low IL-15 concentrations. Model predictions of the threshold requirement for NK cell recruitment to the cell cycle and the subsequent exponential proliferation correlated well with experimental data. In summary, our modeling analysis provides quantitative insight into the regulation of NK cell proliferation at the receptor level and provides a framework for the development of IL-15 based immunotherapies to modulate NK cell proliferation.

## Author Summary

Natural killer (NK) cells are innate immune cells that are important in our bodies' initial defenses against pathogens, like viruses. NK cells rapidly proliferate early during viral infections to provide an expanded pool of effector cells to suppress the infection. This proliferative response is driven by a cytokine called interleukin-15 (IL-15); however, the influence of IL-15 and its receptor (IL-15R) in stimulating NK cell proliferation has not been quantitatively characterized at the cellular level. To better understand the factors controlling the vigorous expansion of NK cells during infections, we developed a mathematical model incorporating IL-15R binding and trafficking parameters that regulate the number of cell-surface IL-15/IL-15R signaling complexes. The analysis of this model provided us with insight on how IL-15-driven NK cell expansion can be modulated through changes in receptor kinetics and expression. Based on model predictions, we were able to draw inferences about NK cell population dynamics and to compare these conclusions with quantitative experimental results. Our results and model have applicability to studies designed to manipulate cell responses in the context of immunotherapies.

## Introduction

Effective immunity against pathogens requires the rapid expansion of lymphocytes capable of an appropriate response. This is illustrated by the substantial numeric increase of murine NK cells early during viral infections [1]–[6]. Vigorous expansion of human NK cells has also been observed during viral infections, including human cytomegalovirus and Hanta virus [7]–[8]. This viral-induced NK cell proliferation is driven by IL-15 and augmented by signaling through NK cell activation receptors that recognize infected cells [2]–[5]. IL-15 not only mediates NK cell proliferation during viral infections but also plays a critical role in NK cell development and homeostasis. Indeed, mice deficient in IL-15 lack NK cells [9] while mice given exogenous IL-15 [5] or that constitutively overexpress IL-15 (e.g., transgenic IL-15 mice [10]) have elevated NK cell numbers. Despite its key role in NK cell physiology, the influence of IL-15 receptor (IL-15R) signaling on NK cell responses (such as proliferation) has not been mechanistically studied and characterized at the cellular level.

The IL-15 receptor is composed of three distinct subunits, α, β, and γ. The β and γ subunits, shared with the IL-2 receptor, bind IL-15 with intermediate affinity (*K_d_* = 10^−9^ M) and mediate IL-15 signaling [11]. The α subunit is specific to the IL-15 receptor, binds IL-15 with high affinity (*K_d_* = 10^−11^ M), and associates with IL-15Rβγ to form the high affinity trimeric IL-15 receptor [12]–[13]. Constitutive expression of IL-15Rβγ is essential for NK cell development and homeostasis. In contrast, IL-15Rα expression is very low on resting NK cells and is upregulated following NK cell activation (e.g., IL-15 stimulation) [3], [14]. IL-15 binding to its receptor activates a number of downstream molecules including Janus kinases and STAT transcription factors as well as Akt, PI(3)K, MAPK kinases, and Ras GTPase, ultimately promoting NK cell development, homeostasis, and proliferation [15]. The contributions of IL-15Rα have been best characterized in the trans-presentation of IL-15 (from cells that make both IL-15 and IL-15Rα to NK cells or CD8 T cells) [16]–[18]; however, a number of studies have implicated a role for IL-15Rα in cis-presentation of IL-15 [19]–[21] or participation in a trimeric complex with IL-15Rβγ on NK cells or CD8 T cells [12], [22].

We have previously examined the NK cell proliferative response to IL-15 at the population level with a two-compartment mathematical model representing quiescent and actively dividing NK cells [23]. Using experimentally derived rate constants, this model was able to accurately predict IL-15-mediated NK cell expansion over time, including changes in NK cell accumulation when IL-15 stimulation was reduced. This approach demonstrated that quiescent and dividing NK cells have distinct division and death rates, which could account for the experimentally observed time delay to first division. Although this modeling approach provided a powerful tool to characterize and understand cytokine-driven proliferation of a population of NK cells, it did not provide mechanistic insight into the regulation of IL-15-stimulated NK cell proliferation at a cellular level. The potential of more mechanistic models to enhance our understanding of the regulation of cellular proliferation is illustrated by several quantitative cellular-level studies focused on IL-2 stimulation of T cells [24]–[28]. These studies demonstrated that the most important parameters regulating IL-2-stimulated T cell proliferation were IL-2 concentration, receptor density, and the surface retention of receptor complexes.

We propose that a mechanistic mathematical model incorporating receptor level kinetics will provide a potent approach to understand the influence of IL-15R signaling on NK cell proliferation and will supplement fundamental understanding garnered from qualitative models [1]–[6] and prior quantitative population-level models [23]. Therefore, we present a mathematical model incorporating IL-15R binding and trafficking parameters that modulate IL-15/IL-15R complex levels. Based on model predictions, we were able to draw inferences about NK cell population dynamics and to compare these conclusions with independent experimental results. Observations from this model provide novel mechanistic insights into the factors regulating IL-15-driven NK cell proliferation including the potential contributions of IL-15Rα upregulation on NK cells in mediating the more rapid proliferation of dividing NK cells.

## Results

### Intermediate affinity binding model

**Figure 1:**
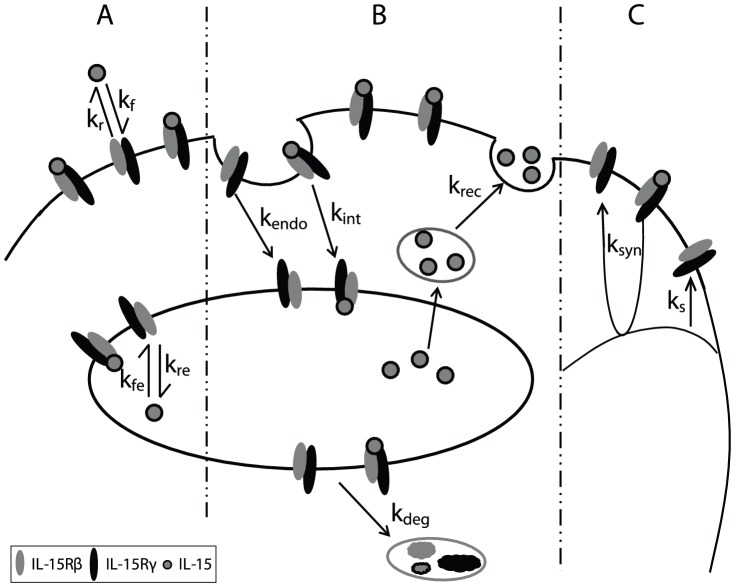
Representative diagram of intermediate affinity IL-15 receptor binding, trafficking and synthesis interactions. Quiescent NK cells constitutively express the β and γ subunits of IL-15R. **A**. Binding: IL-15 binds to receptors on the cell surface with rate *k_f_* and dissociate from IL-15R with rate *k_r_*. Inside the endosome, the on and off rates *k_fe_* and *k_re_* reflect modified binding affinity at lower pH. **B**. Trafficking: Free IL-15 receptors are constitutively internalized with rate k_endo_, and the ligand bound receptor complexes are internalized with rate *k_int_*. The IL-15 ligand recycles from the endosome back to the surface with rate *k_rec_*, and the receptors and complexes are sorted for degradation with rate *k_deg_*. **C**. Synthesis: The IL-15R synthesis is constitutive with rate *V_s_* and can be induced (*k_syn_*) by signaling initiated by the surface complexes.

Quiescent NK cells express IL-15Rβγ, which binds IL-15 with intermediate affinity. To model IL-15 stimulation of quiescent NK cells, we evaluated the parameters that alter IL-15Rβγ and IL-15/IL-15R complex numbers, including ligand binding and dissociation at the cell surface and in endosomes (Fig. 1A), the internalization, recycling, and degradation of ligand, receptors, and complexes (Fig. 1B), and the constitutive and induced synthesis of receptors (Fig. 1C). These parameters and interactions were incorporated into differential equations describing the IL-15 ligand, receptors, and complexes at the cell surface and in endosomes.
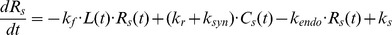


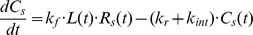


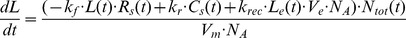


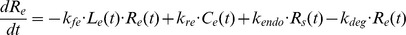


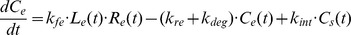


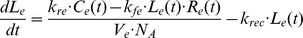



We incorporated a number of simplifying assumptions into these differential equations. First, quiescent NK cells express sufficiently low levels of IL-15Rα [3], [14] that we assumed that IL-15Rα expression on these cells was effectively zero. Second, we assumed that the expression of the common gamma chain was non-limiting on NK cells which allowed us to represent the intermediate affinity IL-15R as IL-15Rβγ heterodimers while ignoring any potential minor contributions of IL-15Rβ homodimers. Third, IL-15Rβ subunits have a cytoplasmic motif targeting internalized receptors to lysosomes [29]–[32], so we assumed that internalized intermediate affinity receptors (*R_e_*) and complexes (*C_e_*) were completely degraded and not recycled back to the cell surface. In contrast, we assumed that free IL-15 in endosomes was recycled back to cell surface with a first order rate constant, k*_rec_*, in a similar manner to IL-2 [29] and other soluble ligands such as transferrin [32]–[33]. (This simplifying assumption minimized ligand depletion in our model which is consistent with observations in our prior in vitro studies [23]. However, ligand depletion might be relevant in some situations, including very low concentrations of IL-15 in small volumes of media in vitro or when increasing the receptor number in silico by substantially modulating the constitutive synthesis rate). Synthesis of IL-15Rβγ was assumed to be both constitutive (*k_s_*) and induced by cell-surface associated IL-15Rβγ signaling (*k_syn_*). Finally, we elected to ignore the spatial impact of trans-presented IL-15/IL15-Ra by stromal or dendritic cells on increasing the local effective concentration of IL-15 since we were evaluating the influence of IL-15 across a wide spectrum of ligand concentrations.

**Figure 2:**
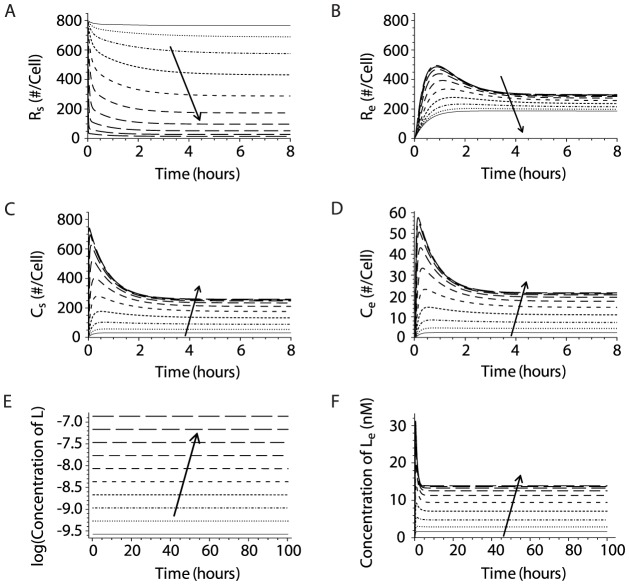
IL-15 concentration quantitatively influences the receptors, complexes and ligands on the surface and in endosomes of quiescent NK cells. Simulations of intermediate affinity receptor binding on NK cells were performed using estimates of kinetic parameters derived from published studies. Solutions of differential equations were depicted in two columns, showing receptor, IL-15/IL-15R complex numbers, and ligand concentration at the cell surface (**A**, **C**, **E**), and in endosomes (**B**, **D**, **F**). The model solutions were obtained from simulations where IL-15 concentration serially doubled from 3.9 ng/ml to 2000 ng/ml, depicted by different lines. The arrow represents increasing IL-15 concentrations.

We solved the system of differential equations for a range of IL-15 concentrations (3.9 ng/ml to 2000 ng/ml) using initial variable values and parameter estimates delineated in Tables 1 and 2. The receptors and complexes at the cell surface and in endosomes rapidly reached steady state following IL-15 stimulation (Figs. 2A–D). As IL-15 concentrations increased, more IL-15 bound to free receptors (Figs. 2A and B), resulting in lower receptor numbers and higher complex numbers both at the surface and in endosomes (Figs. 2C and D). The model predicted that the total number of receptors and complexes on the cell surface rapidly decreased as complexes undergo ligand-induced internalization. This prediction corresponds well with experimental studies that demonstrate a rapid decrease in IL-15Rβ on the cell surface upon incubation with IL-15 (Fig. S1). As the IL-15 concentration approached 2000 ng/ml, the number of surface complexes/cell plateaued at 257/cell (Fig. 2C) as surface receptor binding was saturated (Fig. 2A). At each IL-15 concentration, surface complexes greatly outnumbered those in the endosome at steady state (Figs. 2C and D). Finally, the ligand concentration in the media remained constant (Figs. 2E and F), even at the low IL-15 concentrations. This conclusion was consistent with experimental observations that culturing NK cells in various concentrations of IL-15 for 48 hours did not result in appreciable ligand depletion [23].

**Table 1:** Differential equation variables and initial values.

Parameter	Definition	Initial Value	Reference
**Intermediate affinity IL-15R variables**			
N_tot_	total NK cell number	25,000	[23]
V_m_	cell culture media volume	200 µL	
R_s_	free surface IL-15Rβγ	800	[14]
R_e_	endosomal free IL-15Rβγ	0	
C_s_	surface IL-15/IL-15Rβγ complex	0	
C_e_	endosomal IL-15/IL-15Rβγ complex	0	
L	media intermediate affinity ligand concentration	variable, mole/L	
L_e_	endosomal intermediate affinity ligand concentration	0 mole/L	
**High affinity IL-15R variables**			
C_s_′	surface IL-15/IL-15Rαβγ complex	0	
C_e_′	endosomal IL-15/IL-15Rαβγ complex	0	
Λ_s_	surface high affinity ligand	0	
Λ_e_	endosomal high affinity ligand	0	

Model state variables were used to analyze IL-15R binding, trafficking, and synthesis. The high affinity binding model introduces additional variables as a result of the inclusion IL-15Rα.

**Table 2:** Parameters for intermediate and high affinity binding models.

Parameter	Description	Estimate	Reference
**Shared trafficking parameters**			
V_e_	Total endosomal volume in one cell	10^−14^ L	[47]
k_endo_	Constitutive receptor internalization rate constant	0.42 h^−1^	[48]
k_rec_	Endosomal recycling rate constant	7.4 h^−1^	[33]
k_deg_	Complex and receptor degradation rate constant	2.1 h^−1^	[49]
k_int_	IL-15 receptor complex internalization rate constant	2.4 h^−1^	[48]
**Intermediate affinity IL-15R parameters**			
k_f_	IL-15/IL-15Rβγ association rate constant	9.0·10^8^ M^−1^h^−1^	[46]
k_r_	IL-15/IL-15Rβγ dissociation rate constant	3.6 h^−1^	[46]
k_fe_	IL-15/IL-15Rβγ endosomal association rate constant	2.9·10^−2^ M^−1^h^−1^	Adapted [25]
k_re_	IL-15/IL-15Rβγ endosomal dissociation rate constant	2.9·10^1^ h^−1^	Adapted [25]
k_s_	IL-15Rβγ constitutive synthesis rate constant	3.6·10^2^ h^−1^	Based on [14]
k_syn_	IL-15Rβγ induced synthesis rate constant	1.2 h^−1^	Based on [14]
**High affinity IL-15R parameters**			
k_f_′	IL-15/IL-15Rαβγ association rate constant	1.3·10^9^ M^−1^h^−1^	[34]
k_r_′	IL-15/IL-15Rαβγ dissociation rate constant	5.0·10^−2^ h^−1^	[34]
k_fe_′	IL-15/IL-15Rαβγ endosomal association rate constant	4.0·10^−4^ h^−1^	Adapted [25]
k_re_′	IL-15/IL-15Rαβγ endosomal dissociation rate constant	4.0·10^−1^ h^−1^	Adapted [25]
k_syn_′	High affinity ligand induced synthesis rate constant	1.5 h^−1^	Estimated [14]
ξ_surf_	Conversion factor at cell surface rate constant	1.1·10^−9^ M	Estimated
ξ_endo_	Conversion factor in the endosomal rate constant	1.5·10^−8^ M	Estimated

Shared trafficking parameters and unique binding and synthesis parameters for the intermediate and high affinity IL-15R were obtained or estimated from published experimental measurements. N_A_ represents Avogadro's number.

### Estimate of NK cell fractional recruitment from model predictions of the cell cycle threshold

We hypothesized that the number and duration of IL-15/IL-15R complexes on the surface of a quiescent NK cell must cross a threshold to trigger sufficient downstream signaling to initiate cell division. In our model, we designated the cell cycle threshold as the minimum number and duration of cumulative complexes necessary for NK cell recruitment into the cell cycle. Because IL-15/IL-15R complex numbers rapidly reached steady state (∼2 hrs; Fig. 2C), we made the simplifying assumption that the IL-15 regulated cell cycle threshold was equal to the product of steady state surface complex number and the experimentally determined time delay to first division (*τ*) at low IL-15 concentrations (i.e., area-under-the-curve (AUC) of *C_s_* vs *t* at *t* = *τ*).

**Figure 3:**
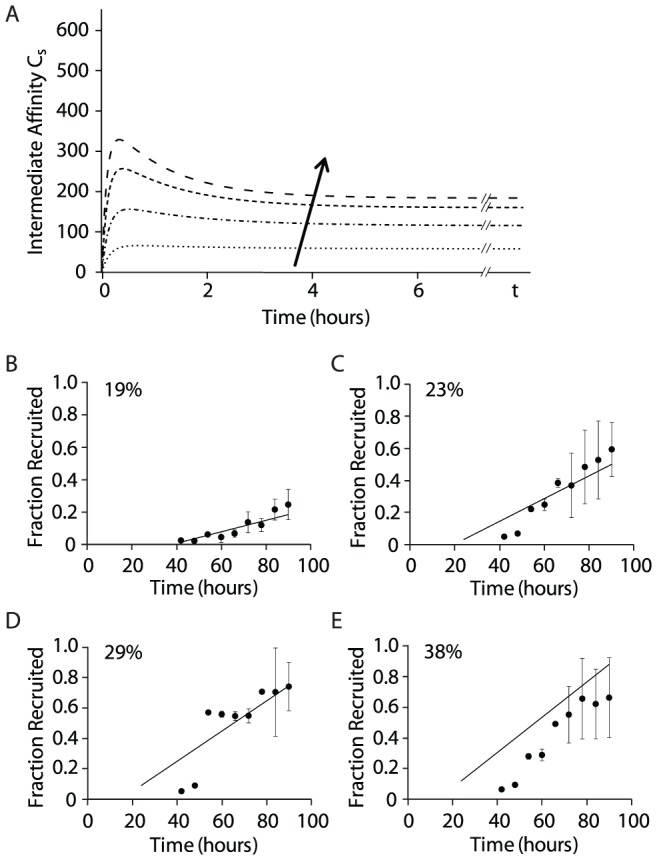
Steady state cell surface complexes determine NK cell recruitment to the cell cycle. **A**. Surface IL-15/IL-15R complex numbers were calculated from model simulations for IL-15 concentrations of 9, 25, 50, and 75 ng/ml and were plotted for *t* hours. The arrow represents increasing IL-15 concentration. **B–E**. The cell cycle threshold (generated *C_s_* from the immediate affinity model) is used to predict the fraction of NK cells recruited to divide at various times. The model predictions (solid lines) are compared with results generated from independent experiments (filled circles) where IL-15 concentrations were 9 ng/ml (**B**, n = 3), 25 ng/ml (**C**, n = 4), 50 ng/ml (**D**, n = 2), and 75 ng/ml (**E**, n = 3). The quality of prediction is represented by the normalized root mean squared deviation (NRMSD). The NRMSD of model prediction vs. experimental data are shown as percentages in the upper left of each graph. For reference, linear regression was performed for all four sets of experimental data, and the NRMSD values of the linear regressions were 12% (9 ng/ml), 5% (25 ng/ml), 19% (50 ng/ml), and 9% (75 ng/ml).

The time delay to first division has been estimated for NK cells stimulated with various concentrations of IL-15 as the time at which the normalized mean division number of dividing NK cells is equal to one [23]. At the lowest concentration at which IL-15 stimulated NK cell proliferation was reliably measured (9 ng/ml), the experimentally determined time delay was 37.8 hours [23]. Our model predicted that stimulation with 9 ng/ml of IL-15 would result in 58 surface complexes/cell at steady state (Fig. 3A). Therefore, we estimated the cell cycle threshold (*C_s_*_,*threshold*_) to be 2198 *C_s_* • h/cell (58.2 surface complexes/cell • 37.8 hours). Assuming this estimate of cell cycle threshold was a reasonable approximation of the minimal cumulative IL-15R stimulation required to initiate cell division, we predicted that increasing IL-15 concentrations would alter the likelihood that an individual NK cell would enter the cell cycle (at times greater than the time delay to first division) by increasing the cumulative number of steady state surface complexes.

This estimation of the cell cycle threshold for an individual NK cell does not directly enable the determination of the probability of NK cell recruitment into cell division. However, we propose that the likelihood that an individual cell will divide can be estimated by comparing the “excess” cumulative IL-15/IL-15R complex stimulation (*C_s_* • *t*) over the *C_s_*_,*threshold*_ at time *t* (designated as the cell cycle momentum) to the IL-15/IL-15R complex stimulation necessary to achieve complete recruitment of a NK cell population. The number of steady state surface IL-15/IL-15R complexes on quiescent NK cells plateaus at 257/cell as IL-15 reaches saturating concentrations (e.g., 2000 ng/ml; Fig. 2C). Using previously described methods [23], we calculated that 64 hours was necessary for complete recruitment of NK cells into cell division following stimulation with 2000 ng/ml of IL-15 (Fig. S2). Therefore, the IL-15R stimulus necessary for all NK cells in a population to be recruited into the cell cycle could be estimated as 16448 *C_s_* • h/cell (257 *C_s_*/cell • 64 h). Using this value, we were able to predict the fractional recruitment of quiescent NK cells into cellular division on a population level following IL-15 stimulation for time *t* by calculating the net positive cell cycle momentum (*C_s_* • *t* – *C_s_*_,*threshold*_) divided by the maximum triggered complexes (16448 *C_s_* • h/cell).

This approach allowed us to directly compare predictions of fractional NK cell recruitment (generated with the intermediate affinity binding model) at various times to experimental results. We performed simulations of NK cells stimulated by 9, 25, 50 and 75 ng/ml of IL-15 and determined that the steady state surface complex numbers were 58, 116, 160, and 184, respectively (Fig. 3A). Using these values, we predicted the fraction of NK cells recruited to divide at various times 
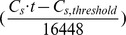
 and compared these predictions with experimental results (Figs. 3B–E). The quality of model predictions was assessed using normalized root mean squared deviation (NRMSD). The NRMSD between model predictions and experimental data was compared with the NRMSD of linear regressions of the experimental data, demonstrating that predictions from our computational model correlated reasonably well with the experimentally derived fractional recruitment of NK cells. The correlation was not as strong at 75 ng/ml, suggesting that this approach may overestimate fractional NK cell recruitment at higher IL-15 concentrations. Estimates of fractional NK cell recruitment using model predictions of time dependent accumulation of cell surface IL-15/IL-15R complexes without the inclusion of the estimated cell cycle threshold did not correlate well with the experimental data (Fig. S3), supporting the definition and use of the proposed cell cycle threshold. These results are consistent with our conclusion that steady state surface complexes function as a surrogate measure of IL-15/IL-15R complex signaling.

### Influence of receptor binding, trafficking, and synthesis on steady state IL-15/IL-15R complex numbers

Based on our initial observations, the number of surface IL-15/IL-15R complexes appears to be a key parameter in regulating the initiation of IL-15-mediated NK cell division. The equation for *C_s_* demonstrates that the interactions that modulate the surface IL-15/IL-15R complex numbers are the binding (*k_f_*), dissociation (*k_r_*), and ligand-induced internalization rate constants (*k_int_*). However, since the number of available free surface receptors also appears in the equation, parameters that affect receptor numbers (including *k_endo_*, *k_s_*, and *k_syn_*) may indirectly influence steady-state complex numbers. Therefore, we varied the value of each of the candidate parameters while keeping the others constant in the simulations to identify the critical parameters that modulate the numbers of surface receptors and complexes.

**Figure 4:**
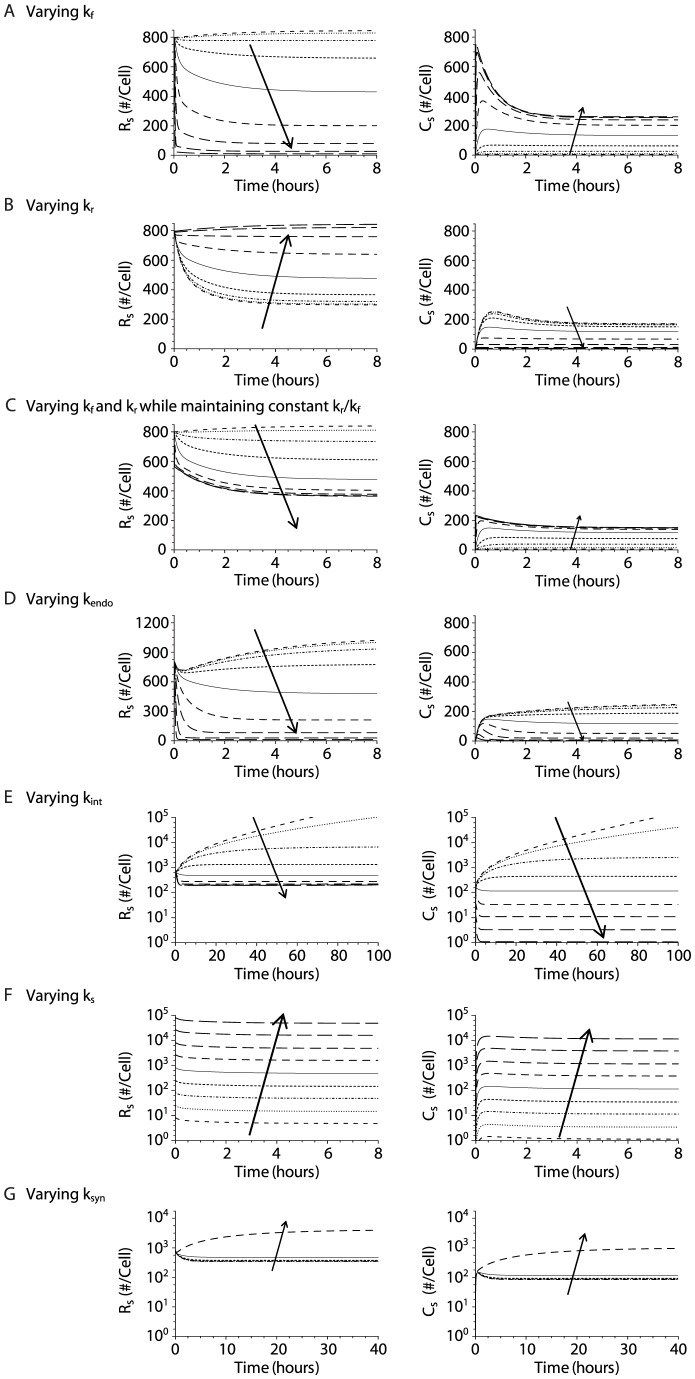
The quantitative influence of intermediate affinity binding model parameters on the steady state cell surface receptor and complex numbers. Model simulations were performed with the value of the parameter of interest varied by a factor of 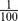, 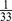, 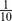, 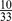, 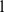, 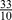, 10, 33, and 100, while the values of all other parameters were held constant. Changes in cell surface receptor and complex numbers as a result of variations in parameter values are shown. Receptor and complex numbers corresponding to different values of the parameter of interest are shown by the dashed curves (with the arrow representing increasing values of the parameter being varied) while the solid curves represent the parameters at their original values. Simulations were performed for model parameters *k_f_* (**A**), *k_r_* (**B**), *k_f_* and *k_r_* (**C**), *k_endo_* (**D**), *k_int_* (**E**), *k_s_* (**F**), and *k_syn_* (**G**) at an IL-15 concentration of 25 ng/ml. Large increases (>10-fold) in *k_syn_* resulted in large perturbations in receptor and complexes numbers (data not shown).

First, we varied the value of *k_f_* (over a range from 0.01 to 100 of the parameter value determined from prior studies) and evaluated the perturbations to the surface receptor and complex numbers. An increase in the binding affinity between IL-15 and IL-15Rβγ significantly reduced the number of free receptors and correspondingly increased the number of surface complexes (Fig. 4A). In contrast, increases in *k_r_* had the opposite effect on *R_s_* and *C_s_* (Fig. 4B). We subsequently investigated varying *k_f_* and *k_r_* at the same time while keeping the equilibrium dissociation constant 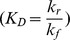 fixed. Under these conditions, the increase in IL-15/IL-15R complexes with increasing *k_f_* was partially blunted (Fig. 4C).

Next we evaluated the influence of the trafficking parameters *k_endo_* and *k_int_*. A decrease in the constitutive internalization rate (*k_endo_*) was associated with an increase in surface receptors as fewer receptors were endocytosed (Fig. 4D). The accumulation of surface receptors resulted in greater formation of surface complexes (Fig. 4D). Conversely, an increase in *k_endo_* resulted in the reduction of steady-state surface receptors and complexes as the receptors were more rapidly internalized to endosomes (Fig. 4D). Decreases in the IL-15-induced internalization rate of IL-15/IL-15R complex (*k_int_*) resulted in greater perturbations of steady state numbers of receptors and complexes than similar changes in *k_endo_* as fewer complexes were internalized (Figs. 4D and E). Indeed, large decreases in *k_int_* (>33 fold) reduced the internalization of complexes to such an extent that the complexes accumulated at the cell surface without coming to steady-state in our model. In contrast, increasing *k_int_* led to decreased surface complexes as complexes were internalized more rapidly (Fig. 4E). The lower steady state levels of surface receptors at high values of *k_int_* occurred as the reduction in surface complex numbers resulted in fewer dissociated complexes and decreased induced synthesis of new surface receptors. However, in contrast to increases in *k_endo_*, increases in *k_int_* led to the reduction but not the depletion of surface receptors.

Finally, we examined the impact of varying either the constitutive or induced receptor synthesis rates (*k_s_* and *k_syn_*) on the steady state number of surface receptors and complexes. Changing the constitutive synthesis rate of receptors, *k_s_*, resulted in corresponding shifts in the steady state receptor and complex levels. In contrast to *k_s_* (a zero-order rate constant), *k_syn_* is a first order rate constant, and increases in *k_syn_* of more than 10-fold caused large perturbations in receptor and complexes numbers.

Interestingly, our analysis suggests that alterations in binding affinity can mimic the impact of changes in IL-15 concentration. For example, an NK cell maximally stimulated by 2000 ng/ml of IL-15 and an NK cell stimulated by 25 ng/ml of IL-15 in the context of a *k_f_* that is increased by 64 fold both maintain 257 surface complexes at steady state. This demonstrates that modifications that result in changes in *k_f_* or *k_r_* could theoretically reduce the cell's dependence on the concentration of IL-15.

### High affinity binding model

We have previously observed that the population-based proliferation rates of dividing NK cells were substantially higher than proliferation rates for quiescent NK cells at various IL-15 concentrations [23]. Although our intermediate affinity binding model demonstrated that sufficient signaling from surface IL-15/IL-15R complexes resulted in quiescent NK cells initiating cell division and enabled the quantitative estimation of NK cell fractional recruitment into the actively dividing subset, it was insufficient to account for the more rapid NK cell proliferation after an NK cell has started to divide. IL-15Rα is expressed on quiescent NK cells at very low levels, but it is upregulated following NK cell activation [3], [14]. We hypothesized that the upregulation of IL-15Rα on NK cells might substantially alter the proliferative response of NK cells by increasing the binding affinity of IL-15 for its receptor. Since surface IL-15/IL-15R complexes served as a surrogate measure of IL-15-mediated signaling, increased surface complexes might be sufficient to account for the more rapid proliferation of dividing NK cells. Therefore, we modified our model to incorporate the upregulation of IL-15Rα on NK cells to evaluate whether this alteration was sufficient to account for the differences in proliferation dynamics between quiescent and dividing subsets of the NK cells.

**Figure 5:**
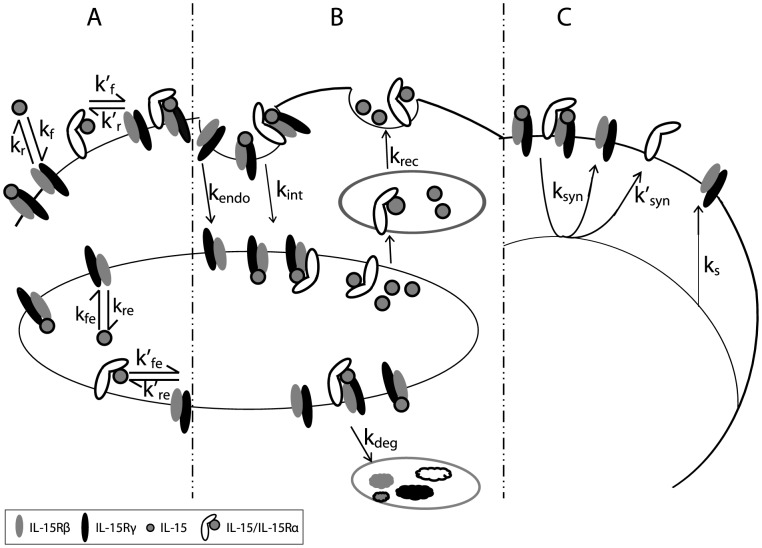
Representative diagram of intermediate and high affinity IL-15 receptor binding, trafficking and synthesis interactions. Activated NK cells upregulate the expression of the high affinity α subunit of IL-15R. **A**. Binding: IL-15Rβγ associates with IL-15 with on and off rates *k_f_* and *k_r_* at the cell surface and *k_fe_* and *k_re_* in endosomes. All IL-15Rα are assumed to rapidly bind IL-15, forming the high affinity ligand for IL-15Rβγ. IL-15/IL-15Rα binds IL-15Rβγ with on and off rates *k_f_^′^* and *k_r_^′^* at the cell surface and *k_fe_^′^* and *k_re_^′^* in endosomes. **B**. Trafficking: Unbound IL-15Rs are constitutively internalized with rate *k_endo_*. IL-15/IL-15Rβγ and IL-15/IL-15Rαβγ complexes are internalized with rate *k_int_*. Soluble IL-15 and the high affinity ligand (IL-15/IL-15Rα) in the endosome recycle to the surface with rate *k_rec_*. Intermediate and high affinity complexes are sorted for degradation with rate *k_deg_*. **C**. Synthesis: The constitutive synthesis of IL-15Rβγ is represented by *V_s_*. *C_s_* and *C_s_′* induce the synthesis of IL-15Rβγ with rate *k_syn_* and the synthesis of IL-15Rα with rate *k_syn_^′^*.

Our initial modeling simulations demonstrated that IL-15 was in excess even at low ligand concentrations and that IL-15 depletion was minimal (Fig. 2E–F). These observations coupled with the extremely high binding affinity of IL-15Rα for IL-15 [13], [34] were sufficient to justify the simplifying assumption that all IL-15Rα molecules were bound to IL-15 [18] and that the IL-15/IL-15Rα complexes on the cell surface or in the endosome did not dissociate [16]–[18]. This assumption allowed us to mathematically represent IL-15/IL-15Rα complexes as “ligands” (*Λ_s_*) capable of binding to IL-15Rβγ with high affinity (Fig. 5A). Thus, our high affinity binding model incorporated the binding of both intermediate and high affinity ligands with distinct binding and dissociation kinetics at the cell surface and in endosomes (Table 2). Because both ligands bind IL-15Rβγ and IL-15Rα has a very short cytoplasmic tail [13], we assumed that the receptor complexes share identical trafficking parameters (Fig. 5B). Following internalization with the rate constant *k_int_*, we assumed that both complexes (*C_s_* and *C_s_′*) were sorted for degradation with the rate constant *k_deg_*. Based on the similarity between IL-15Rα and IL-2Rα, we assumed that the degradation of IL-15Rα not associated with IL-15Rβγ was negligible [29]. Since IL-15Rα has been shown to recycle back to the cell surface bound to IL-15 [16]–[17], we made the simplifying assumption that the high affinity ligand (IL-15/IL-15Rα) recycles to the cell surface with the rate constant *k_rec_*. In addition, we assumed that common signaling pathway between *C_s_* and *C_s_′* induces the synthesis of IL-15Rβγ and IL-15Rα with rates *k_syn_* and *k_syn_′*, respectively (Fig. 5C).

In light of these assumptions, we modified the previous model by incorporating variables and parameters (Table 2) associated with the high affinity ligand, IL-15/IL-15Rα, into the following system of differential equations.
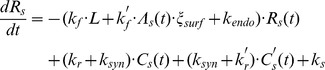


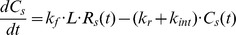


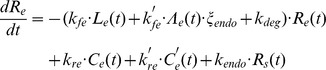


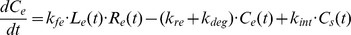


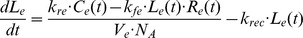


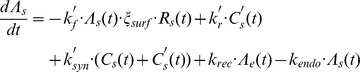


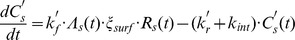


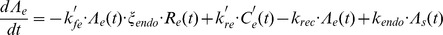


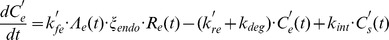



**Figure 6:**
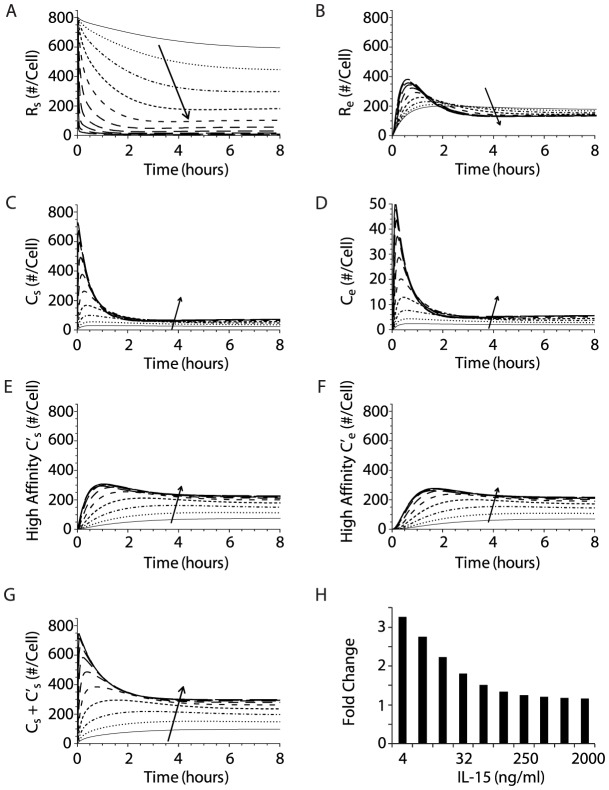
The upregulation of IL-15Rα amplifies IL-15R signaling and modulates the steady state numbers of receptors, complexes, and ligands on the cell surface and in endosomes of dividing NK cells. Numerical solutions of the computational model are depicted in two columns, showing receptor, intermediate affinity complex, and high affinity complex at the cell surface (**A**, **C**, **E**) and in endosomes (**B**, **D**, **F**). Model solutions were obtained from simulations where IL-15 concentration serially doubled from 3.9 ng/ml to 2000 ng/ml, represented by different lines with the arrow denoting increasing IL-15 concentration. **G**. The total number of signaling complexes at the cell surface is shown as the sum of intermediate and high affinity complexes. **H**. Fold change in total steady state cell surface IL-15/IL-15R complex numbers on dividing cells (with upregulation IL-15Rα) compared with quiescent NK cells (which express no appreciable IL-15Rα). The fold changes in this ratio at different IL-15 concentrations (serially doubled from 3.9 to 2000 ng/ml) are depicted by solid bars.

When we evaluated simulations of the high affinity binding model at various IL-15 concentrations, several key differences in steady state numbers of surface receptors and complexes were evident in comparison with the intermediate affinity binding model, reflecting the increased binding of the high affinity ligand (i.e., IL-15/IL-15Rα). The steady state numbers of unbound surface receptors (IL-15Rβγ) were decreased at lower IL-15 concentrations in the high affinity binding model compared with the intermediate affinity binding model (Fig. 6A). In contrast, there were substantially more internalized receptors in the high affinity binding model (Fig. 6B). *C_s_* and *C_e_* were reduced in comparison to values in the intermediate affinity model as a greater proportion of receptors were occupied by the high affinity ligand (Figs. 6C and D). High affinity complexes (*C_s_′*) outnumbered intermediate affinity complexes (*C_s_*) on the cell surface by a factor of approximately 3 at all IL-15 concentrations (Figs. 6C and E). Moreover, substantial numbers of high affinity complexes accumulated in the endosome following internalization (*C_e_′*), due to both ligand-induced internalization of *C_s_′* and less dissociation of the high affinity ligand in the low pH endosomal environment (Fig. 6F). However, the most striking difference was that the inclusion of IL-15Rα on the NK cell substantially raised the total number of steady-state surface complexes (Fig. 6G). Assuming that the cumulative number of surface complexes (both *C_s_* and *C_s_′*) was proportional to the magnitude of IL-15 mediated signaling, IL-15Rα on NK cells amplified the receptor signal much more at lower IL-15 concentrations (Fig. 6H), while higher IL-15 concentrations appeared to mask the impact of IL-15Rα on NK cells due to more effective intermediate affinity receptor binding at higher IL-15 concentrations.

The inclusion of IL-15Rα in the high affinity binding model modified the sensitivity of the model to changes in trafficking and synthesis parameters (Fig. S4). Large increases in the IL-15-induced internalization rate (*k_int_*) in both the intermediate and high affinity models were predicted to drive the total complexes on the cell surface to negligible levels (Figs. 4E and S3A). However, in contrast to the elevated steady state receptor numbers observed in the intermediate affinity binding model sensitivity analysis, decreased *k_int_* values led to depletion of free surface receptors as the increased numbers of surface complexes (Fig. S4B) stimulated the upregulation of the high affinity ligand, *Λ_s_*. Similarly, increasing the induced synthesis rate of the high affinity ligand (*k_syn_′*) markedly decreased the numbers of surface receptors while increasing the total number of surface complexes (Fig. S4D). In contrast, large decreases in *k_syn_′* mimicked the intermediate affinity model predictions of receptor and complex numbers as the generation of the high affinity ligand was substantially reduced. Even in the context of the high affinity binding of IL-15 and IL-15Rαβγ, this parameter sensitivity analysis demonstrates that the NK cell proliferation response could potentially be further modulated by molecular or biochemical manipulations of the internalization and synthesis of the intermediate and high affinity IL-15Rs.

### Formation of high affinity complexes facilitates exponential expansion of dividing NK cells

To quantify the magnitude of the NK cell proliferative response to IL-15, we measured ^3^H-thymidine incorporation in NK cells incubated in various concentrations of IL-15 for 72 hours (Fig. S5A). We plotted the thymidine values at each IL-15 concentration as a fraction of the maximum thymidine incorporation (observed at 2000 ng/ml IL-15). Above IL-15 concentrations of 7.8 ng/ml, the response increased exponentially until plateauing at values greater than 125 ng/ml, suggesting that NK cell surface receptors were nearing saturation at this IL-15 concentration.

**Figure 7:**
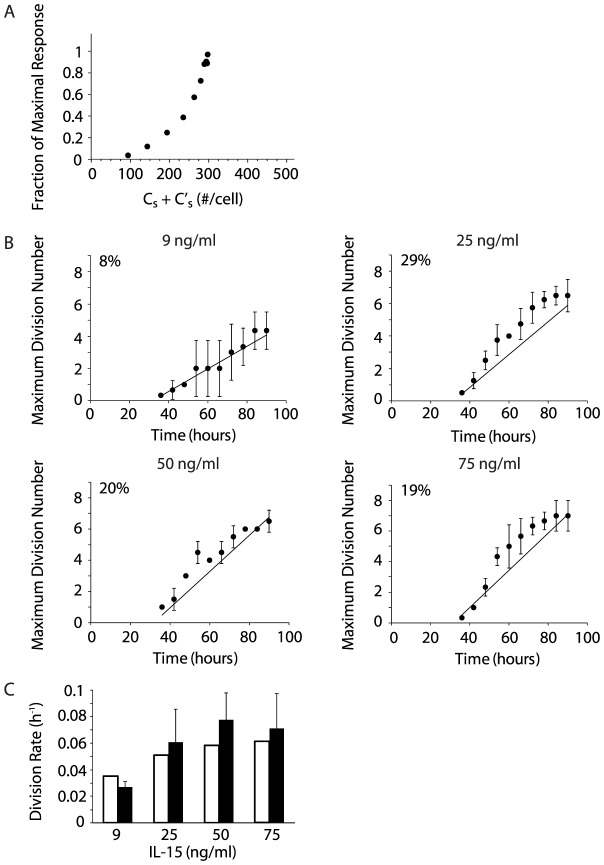
Formation of high affinity receptor-ligand complexes facilitates exponential expansion of dividing NK cells. **A**. The total steady state cell surface IL-15/IL-15R complex numbers in the high affinity binding model stimulate an exponential proliferative response, illustrated by plotting the fraction of maximal response vs. total *C_s_* at the corresponding IL-15 concentrations. **B**. The maximum number of divisions calculated from the time elapsed since recruitment to cell division by the interdivision time for four different IL-15 concentrations. Solid lines represent model predictions, and filled circles represent the maximum detectable number of NK cell divisions obtained from independent experiments (9 ng/ml n = 3, 25 ng/ml n = 4, 50 ng/ml n = 2, and 75 ng/ml n = 3). The quality of prediction is represented by the normalized root mean squared deviation (NRMSD). The NRMSD of model prediction vs. experimental data are shown as percentages in the upper left of each graph. For reference, linear regression was performed for all four sets of experimental data, and the NRMSD values of the linear regressions were 9% (9 ng/ml), 10% (25 ng/ml), 14% (50 ng/ml), and 12% (75 ng/ml). **C**. Population mean division rate is estimated from the interdivision time for four IL-15 concentrations. White bars represent model predictions while black bars represent experimental data from the analysis of NK cell populations in 2–4 independent experiments.

To determine the functional dependence of NK cell division on IL-15-mediated signaling, we used cell surface IL-15/IL-15R complexes as a surrogate for IL-15 receptor signaling and transformed the abscissa of Figure S5A from IL-15 concentration to the corresponding steady state total surface complex numbers calculated in our high affinity binding model (Fig. 7A). This approach builds on previous studies modeling EGF-mediated fibroblast proliferation and IL-2-stimulated T cell division [24], [35]. The increase in surface complex numbers (as the IL-15 concentration was increased) was associated with an exponential increase in the proliferation (i.e., thymidine incorporation) of activated NK cells until surface complex numbers reach saturation at 298 complexes/cell (Fig. 7A). These findings suggest that NK cells expand exponentially after starting to divide and provide additional support for our hypothesis that the magnitude of the NK cell proliferative response is dependent on the total number of steady state surface complexes

In the intermediate affinity binding model, we determined that a threshold of 2198 *C_s_* • h/cell must be reached to initiate a sufficient number of downstream molecular interactions to initiate NK cell division. Assuming that this cell cycle threshold is also applicable to actively dividing NK cells, the cell cycle threshold and the steady state surface complex numbers from the high affinity binding model allow the calculation of interdivision time (minimal time to complete one cell division) after a cell begins to divide and the estimation of division rates at different IL-15 concentrations. We hypothesized that the increased number of surface complexes on actively dividing NK cells would allow them to traverse the cell cycle threshold in less time. We divided the cell cycle threshold by the total steady state surface complex numbers predicted in the high affinity binding model to calculate the interdivision time 
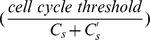
 at four representative IL-15 concentrations (Table 3). Knowing the interdivision time enabled us to estimate the maximum number of times that an NK cell could have potentially divided during a fixed time period, 
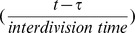
. We made the simplifying assumption that the time delay (*τ*) of 32 hours is independent of IL-15 concentration based on previous experimental NK cell studies [23]. We determined the maximum number of cell divisions following stimulation with four representative IL-15 concentrations for 90 hours and compared these predictions with experimental results (Fig. 7B). (The caveat in the experimental studies is that maximal cell divisions at high IL-15 concentrations at later times may be underestimated given that tracking cell division with CFSE is limited to 7 or 8 divisions as the CFSE is diluted with each cell division). Our model's predictions accounted for the experimentally observed maximal number of NK cell divisions. This strong correlation supports our hypothesis that the cell cycle threshold is an inherent cellular parameter that regulates NK cell proliferation.

**Table 3:** Estimates of NK cell interdivision times and division rates.

[IL-15] (ng/ml)	Surface Complex (#/cell)	Interdivision Time (h)	Mean Division Rate (10^−2^ h^−1^)
9	154	14.2	3.5
25	224	9.8	5.1
50	256	8.6	5.8
75	270	8.2	6.1

Steady-state surface complex numbers from model solutions were used to calculate interdivision time and subsequently the mean division rate of NK cells stimulated by 9, 25, 50 and 75 ng/ml of IL-15.

The mean division rate of a population of NK cells provides a more comprehensive assessment of population dynamics than the estimate of the maximum number of cell divisions. We determined the division rate of the fastest dividing cohort of NK cells from the inverse of the interdivision time. However, cells proceed through multiple cell divisions asynchronously. Based on previously published experimental data [23], we made the simplifying assumption that the distribution of dividing NK cell cohorts was approximately Gaussian with regard to division progression (Fig. S6). Therefore, we reasoned that the division rates within a population of NK cells varied in roughly a Gaussian distribution from a maximum value (represented by the inverse of the interdivision time) to very low values for cells that have divided only once (near zero at times >2*τ*). The mean division rate represents the mean value of this Gaussian distribution estimated as 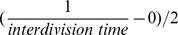. Mean division rates calculated from the interdivision times at several different IL-15 concentrations (Table 3) were compared with experimentally derived division rates (Fig. 7B). The reasonably good correlation of the predicted mean division rates and the experimentally derived division rates provides support for our hypothesis that the upregulation of IL-15Rα on NK cells amplifies the stimulatory signal of IL-15, facilitating the exponential expansion of the NK cell population.

## Discussion

To quantitatively investigate IL-15-mediated NK cell proliferation at a cellular level, we developed a mathematical model incorporating IL-15R binding and trafficking parameters that modulate IL-15/IL-15R complex levels. Based on model predictions, we were able to draw inferences about NK cell population dynamics and compare these conclusions with experimental results. This approach, leveraging predictions from a mechanistic IL-15R model to make inferences about NK cell behavior on a population level, provided a number of unique insights into the regulation of IL-15-stimulated NK cell proliferation: 1) IL-15/IL-15R complexes on the cell surface are a key determinant of the magnitude of the IL-15 proliferative signal and function as an effective surrogate measure of IL-15R signaling, 2) the cumulative number and duration of IL-15/IL-15R complexes appear to cross a quantitative threshold prior to initiation of NK cell division, 3) upregulation of IL-15Rα on dividing NK cells substantially increases the number of total cell surface IL-15/IL-15R complexes, resulting in an increased division rate, and 4) high affinity binding mediated by IL-15Rα on NK cells particularly enhances cellular responses at low IL-15 concentrations.

In the development of our model, we hypothesized that surface IL-15/IL-15R complexes mediated IL-15 signaling. This hypothesis was based on prior computational and experimental studies in other systems that implicated surface complexes as the critical parameter in determining cellular responses with internalization attenuating receptor-mediated signaling [24], [35]–[41]. For example, fibroblasts transfected with an internalization defective EGF receptor mutant required 10-fold less EGF to stimulate a half maximal mitogenic response [35]–[36]. Similarly, in the type 1 angiotension (AT1) system, overexpression of an accessory protein (ATRAP) increased the internalization of AT1 receptor-angiotension II complexes resulting in decreased phosphorylation of STAT 3 and Akt and inhibition of DNA synthesis [39]. Furthermore, previous studies of the analogous IL-2 system in T cells have also focused on signaling and regulation of IL-2 receptor interactions at the cell surface [24], [27], [40]. In our work, the utility of steady state surface IL-15/IL-15R complexes in determining the cell cycle threshold and the interdivision time demonstrated that steady state cell surface IL-15/IL-15R complexes were an effective surrogate measure of IL-15R signaling. Indeed, when we modified the calculations to compare cell surface complexes, endosomal complexes, or a combination of both as surrogate measures of IL-15R signaling, the addition of endosomal complexes did not improve the correlation with experimental fractional recruitment data and resulted in worse correlation with experimental data on maximum division number (data not shown).

Our work suggests that NK cells accumulate a sufficient number of IL-15/IL-15R complexes over time before initiating cell division (i.e., cell cycle threshold). The concept of a threshold necessary to begin cell division has previously been proposed for IL-2 stimulation of T cell proliferation [28], [42]. Indeed, Fallon and colleagues [24] reported that the growth rate of T cells was minimal below a threshold number of IL-2/IL-2R complexes per cell and plateaued when surface receptors were saturated. Using predictions of steady state cell surface IL-15/IL-15R complex numbers from the intermediate affinity binding model with an experimentally observed time delay to first division at a low IL-15 concentration, we were able quantitatively define the cell cycle threshold for 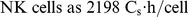 ( i.e., area-under-the-curve (AUC) of C*_s_* vs *t* when *t* = *τ*). We utilized this cell cycle threshold to make predictions of fractional recruitment of quiescent NK cells into the dividing population, maximum division numbers of NK cells stimulated with different IL-15 concentrations, and mean NK cell division rates. The correlation of these predictions with experimental results corroborated our approach to quantify the cell cycle threshold necessary for IL-15-stimulated NK cell proliferation as well as our hypothesis that the cell cycle threshold is an intrinsic parameter in both quiescent and dividing subpopulations of NK cells and is independent of division number.

After crossing the threshold necessary to initiate cellular division, NK cells proliferate more rapidly in subsequent divisions. Indeed, population-based proliferation rates calculated for dividing NK cells at various IL-15 concentrations were approximately three times higher than those for quiescent NK cells [23]. Our intermediate affinity binding model facilitated the quantitative estimation of the cell cycle threshold necessary for quiescent NK cells to initiate cell division and of NK cell fractional recruitment into the actively dividing subset; however, it was insufficient to account for more rapid proliferation after an NK cell has started to divide. An evaluation of the impact of various binding, trafficking, and synthesis parameters on steady state numbers of surface IL-15/IL-15R complexes illustrated that increasing the binding affinity of IL-15 for its receptor resulted in higher numbers of surface complexes. One way that the effective binding affinity of IL-15 to IL-15Rβγ can be physiologically modified is through the upregulation of IL-15Rα on NK cells following IL-15 stimulation. The incorporation of IL-15Rα upregulation on NK cells into our model resulted in significantly higher numbers of steady state cell surface IL-15/IL-15R complexes and was sufficient to account for the more rapid proliferation of dividing NK cells. The maximum division number and mean division rate, calculated with the total cell surface IL-15/IL-15R complexes (*C_s_*+*C_s_*′) from the high affinity binding model, correlated well with experimental results. The impact of the upregulation of IL-15Rα on NK cells was greatest at low IL-15 concentrations, with higher IL-15 concentrations masking the influence of IL-15Rα due to increased formation of intermediate affinity receptor complexes.

Given the substantial structural similarities between IL-15 and IL-2 quaternary complexes, differences in IL-2 and IL-15 receptor signaling have been attributed to the much higher affinity binding of IL-15 to IL-15Rα (*K_D_* = 38 pM) in comparison to the binding affinity of IL-2 to IL-2Rα (*K_D_* = 4.8 nM) [22], [34]. IL-2Rα captures IL-2 at the cell surface, enriching the surface-associated cytokine and facilitating IL-2 binding to the intermediate receptor (IL-2Rβγ). In contrast, the higher affinity of IL-15Rα for IL-15 (with a substantially lower off rate of 0.05 h^−1^ compared to the *k_r_* = 72 h^−1^ for IL-2 from IL-2α) results in greater formation and persistence of IL-15/IL-15Rα complexes. The best characterized role of IL-15Rα is in trans presentation (from cells that make both IL-15 and IL-15Rα) resulting in tight control of IL-15 localization and effectively increasing the local concentration of IL-15 that an NK cell or a CD8 T cell experiences [16]–[18]. Although IL-15Rα is strongly upregulated on NK cells following activation [3], [14], few studies have focused on the contributions of cis presentation of IL-15 by IL-15Rα [19]–[21]. Given its very high affinity for its ligand, we predict that IL-15Rα on NK cells would be more effective at sequestering IL-15 on the cell surface than IL-2Rα is at enriching IL-2, particularly at low ligand concentrations. Indeed, our model illustrates that IL-15Rα on NK cells amplifies IL-15R signaling to a greater extent at low IL-15 concentrations. Based on the conclusions from our model, we predict that IL-15 stimulated proliferation of NK cells that lack the ability to upregulate IL-15Rα will be less vigorous than the proliferation observed in wild type NK cells.

Our work with IL-15 and its receptor on NK cells builds on the pioneering experimental studies of Cantrell and colleagues [27] and prior computational modeling of the analogous IL-2 system in stimulating T cells [24]. These studies established that IL-2 concentration, receptor density, and the duration of receptor-ligand interactions were critical factors in IL-2 stimulated T cell proliferation. More recent computational studies [40], which focused on the binding kinetics of IL-2 to IL-2Rα and subsequent interaction with IL-2Rβγ over short time periods (e.g., 10 min), demonstrated that the density of IL-2R subunits modulated the sensitivity of the cell's response to IL-2. This work provided novel insight into differences in IL-2 responses between effector T cells and regulatory T cells (which constitutively express IL-2Rα). Although IL-2 and IL-15 share the same intermediate affinity receptor (IL-2/IL-15Rβγ), there are a number of differences between these cytokines including distinct individual high affinity receptors, affinity of IL-2 and IL-15 for their receptor subunits (discussed in the previous paragraph), differences in constitutive expression of the receptor subunits (e.g., NK are dependent on IL-15 and constitutively express high levels of IL-2/IL-15Rβ), and T cell autocrine production of IL-2. Despite these differences, we were able to utilize insights gained from previous experimental and computational IL-2 studies in our evaluation of the impact of IL-15 receptor binding, trafficking, and synthesis parameters on NK cell proliferation.

The quantitative insights provided by mechanistic modeling of IL-15 and IL-15R interactions on a cellular level combined with computational analysis of NK cell population studies suggests that it may be possible to therapeutically manipulate the interaction of IL-15 and IL-15R to modulate NK cell responses in clinically relevant situations, such as intractable viral infections, cancers, or NK cell lymphoproliferative disorders. Recent studies have demonstrated the potential to modulate cellular proliferation via alterations in cytokine binding or receptor trafficking. For example, engineered IL-2 molecules optimized for binding to either IL-2Rα or IL-2Rβ exhibited enhanced IL-2R signaling [43]–[44] while deficiencies in EGF receptor internalization resulted in increased cellular proliferation [35]. Predictions from our model will not only guide the development of novel therapeutic strategies to modulate IL-15R signaling but also provide testable hypotheses for future experiments including studies of NK cells with mutations or deficiencies in IL-15Rα.

## Materials and Methods

### Mice

Female C57BL/6 (B6) mice were obtained from the National Cancer Institute (Charles River, MA). They were maintained under specific pathogen-free conditions and used between 8 and 16 weeks of age. All experiments were conducted in accordance with institutional guidelines for animal care and use based on the Guide for the Care and Use of Laboratory Animals of the National Institutes of Health. The protocol was approved by the Animal Studies Committee at Washington University (#20110104).

### Proliferation assays

Murine splenocytes were enriched for NK cells via negative selection (Miltenyi, CA) and cultured with murine IL-15 (Peprotech, NJ) as previously described [23]. CFSE-labeled splenocytes were plated in 96-well plates (2.5×10^4^ NK cells/well) and cultured for 4 days in 200 µl of growth media (RPMI 1640 medium supplemented with 10% fetal calf serum) containing various concentrations of murine IL-15 (PeproTech, Rocky Hill, NJ). Cells were harvested at different time points and analyzed by flow cytometry. The number of dividing NK cells and their proliferation rate were determined using precursor cohort analysis and the two compartment model analysis [23].

Thymidine incorporation assays were performed with B6 splenocytes as previously described [23], [45]. Briefly, NK cells from B6 splenocytes were enriched by passage over nylon wool columns and cultured with murine IL-15 (Peprotech, United Kingdom) at concentrations ranging from 1 ng/ml to 2000 ng/ml (0.067 nM to 133 nM) for 72 hours. After 48 hours, ^3^H-thymidine (0.4 µCi/well; Perkin-Elmer, MA) was added. Incorporated ^3^H-thymidine was measured with a liquid scintillation counter (Wallac; Gaitherburg, MD).

### Estimate of the maximum number of NK cell divisions

Flow cytometry analysis was performed with a FACScalibur flow cytometer (BD Pharmingen) to detect CFSE dilution in NK cells stimulated by 9, 25, 50, or 75 ng/ml (0.60, 1.67, 3.33 or 5.00 nM) of IL-15 at various times. These concentrations of IL-15 were chosen based on experimental thymidine studies of NK cell proliferation (Fig. S5A) and CFSE-based studies of NK division rates (Fig. S5C) which demonstrated that the linear range around the EC_50_ value on a semi-log response curve (representing the range of concentrations over which changes in the stimulus resulted in meaningful changes in response) was from ∼8 ng/ml to 125 ng/ml. Above 125 ng/ml, thymidine incorporation plateaued out at maximal values, while the division rate plateaued at concentrations above 50 ng/ml. These values correlated well with the plateauing of the steady state surface complexes predicted by the high affinity model at IL-15 concentrations greater 75 ng/ml (Fig. S5B). OriginPro 7.5 was used to distinguish the CFSE peaks representing cell cohorts that divided the same number of times. The fraction of the population in each cohort was determined from the ratio of area under each CFSE peak to the total area under the curve. The maximum number of divisions that NK cell have completed was determined from the total number of CFSE peaks.

### Modeling parameter estimation

To account for IL-15R and IL-15/IL-15R complex trafficking, we utilized previously published estimates of *k_endo_*, *k_int_*, and *k_deg_* of IL-2/IL-15βγ in our model. We assumed that *k_rec_* of IL-15 and the IL-15/IL-15Rα complex from endosomes were similar to the recycling of transferrin, given that endosomal sorting of fluid phase components (or membrane associated components that are not retained in the endosome) is relatively ligand independent (with the exception of steric issues with large ligands). In addition, we calculated the ligand concentration inside endosomes using published estimates of the total endosomal volume (*V_e_*) of fibroblasts based on the simplifying assumption that endosomes of mammalian cells have similar volumes.

The intermediate and high affinity binding models share the same trafficking parameters but differ in the binding and synthesis parameters. For the intermediate affinity ligand-receptor binding, we utilized the on and off rate constants of human IL-15/IL-15Rβγ binding for the estimates of *k_f_* and *k_r_* [46]. Estimates of *k_re_* were made based on the studies of IL-2 binding that found that the off rate of IL-2 from its receptor was 8 times higher in the low pH environment of the endosome than on the cell surface [25]. We also assumed that the lower pH in the endosome increased the equilibrium dissociation constant, *K*_d_, of IL-15 in a similar manner to the IL-2 receptor system and calculated *k_fe_* based on our estimate of *k_re_*. Furthermore, varying the estimates of *k_fe_* and *k_re_* by one order of magnitude did not significantly change the solutions of the model parameters (data not shown). At steady state, the number of intermediate affinity surface receptors on unstimulated NK cells was determined by endocytosis and constitutive receptor synthesis. Thus, we estimated *k_s_* from *k_endo_* of IL-2Rβγ based on the observation that approximately 800 intermediate affinity receptors were found on unstimulated NK cells [14]. The induced synthesis rate constant of the intermediate receptors, *k_syn_*, was derived from studies of human NK cells stimulated with IL-15 in vitro [14].

The high affinity ligand-receptor binding describes the association between IL-15 and IL-15Rαβγ. IL-15Rαβγ's high affinity for IL-15 matches that of IL-15Rα, whereas IL-15Rβγ has only intermediate affinity for IL-15 [12]. Therefore, we made the simplifying assumption that the high affinity IL-15 ligand-receptor binding and dissociation rate could be approximated by the interaction between IL-15 and IL-15Rα. To simplify the computational analysis, we assumed that all available IL-15Rα were bound to IL-15, based on the excess of IL-15 and the very high affinity of IL-15 binding to IL-15Rα [18]. Therefore in our model, uniform binding of IL-15Rα and IL-15 results in the formation of a high affinity ligand, *Λ*. We modeled the formation of the quaternary complex of IL-15/IL-15Rαβγ as the association between the high affinity ligand (*Λ*) and the intermediate affinity IL-15R (IL-15Rβγ). Therefore, we estimated *k_f_*′ and *k_r_^′^* of *Λ* for IL-15Rβγ from the binding between IL-15 and recombinant IL-15Rα [34], and the synthesis of IL-15Rα essentially represented the synthesis of the high affinity ligand. We estimated the synthesis rate constant of IL-15Rα from the fold increase under IL-15 stimulation in comparison to basal levels [14]. Variations in the value of *k_syn_′* over one order of magnitude did not significantly affect receptor occupancy. To quantitatively analyze ligand-receptor binding in the high affinity binding model, we made the simplifying assumption that the IL-15/IL-15Rα complexes were evenly distributed on the cell surface and the interior of endosomes. The cellular and the endosomal membrane were approximated as spherical shells with thickness equal to that of a lipid bilayer. We utilized the volumes of the cellular and endosomal membrane to compute conversion factors *ξ_surf_* and *ξ_endo_*, which enabled us to quantify the spatial distribution of IL-15/IL-15Rα complexes. Based on the assumption that the affinity between the high affinity ligand (*Λ*) and the intermediate receptor decreases in lower pH environments, the association and dissociation rate constants of the high affinity ligand in the endosome, *k_fe_^′^* and *k_re_^′^*, were estimated in a similar manner as were *k_fe_* and *k_re_* of the intermediate affinity receptor.

### Model simulation and determination of the accuracy of model predictions

The solutions to the ODEs were obtained from numerical simulations performed using Mathematica 7.0 (Wolfram Research, Champaign, IL). The quality of the model predictions were assessed by computing the normalized root mean square deviation (NRMSD) between model computations and observed experimental values. The sum of squared deviations (SSD) at different time points were computed, and NRMSD was calculated as a percentage value by the following formula:
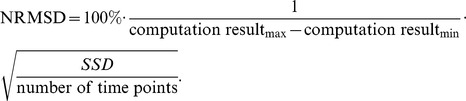
A NRMSD value of 0% indicated absolute agreement between model computations and experimental results. Greater differences between model simulations and experimental data were reflected in higher NRMSD values.

## Supporting Information

Figure S1 **Receptor trafficking kinetics of IL-15 stimulated NK cells.** IL-2/IL-15Rβ expression on NK cells was determined and quantified at different time points following IL-15 stimulation. C57BL/6 splenocytes were plated at 200,000 cells/well in 96 well round bottom plates in the presence or absence of IL-15. PE-conjugated anti-CD122 (TM-β 1) or isotype control Ab were used to determine CD122 expression. The mean fluorescence intensity (MFI) of the isotype control Ab was subtracted from the MFI of the anti-CD122 Ab to determine the MFI of specific CD122 expression. QuantiBrite PE beads (BD Biosciences, San Diego, CA) were used to estimate the antibodies bound per cell. **A**. The number of surface IL-2/IL-15Rβ was shown at 15 min, 30 min, 1 hour, 2 hours, 4 hours, 8 hours and 10 hours after the initiation of stimulation by IL-15 at concentrations 3, 9, 25, 75. Unstimulated samples were used as controls. Data shown is representative of two independent experiments. **B**. NK cells were incubated on ice to inhibit internalization for 1, 2, 4, 6 and 8 hours in the presence or absence or IL-15 (25 ng/ml) and the number of surface IL-2/IL-15Rβ was determined.
(EPS)

Figure S2 **Complete recruitment of quiescent NK cells under saturating IL-15 stimulation.** Numerical simulations using population kinetic parameters [23] were used to estimate the decrement of quiescent NK cells stimulated by 2000 ng/ml of IL-15. The functional dependence of division and death parameters on IL-15 was determined from independent experiments with IL-15 concentrations of 3, 25, or 75 ng/ml [23]. Adopting logarithmic functions to model parameter dependence on the concentration of IL-15, we extrapolated parameter values at 2000 ng/ml and simulated NK cell recruitment at different times. The number of undivided NK cells decreased to 1% of its initial value at 64 hours.
(EPS)

Figure S3 **Alternative model of fractional recruitment without incorporating cell cycle threshold.** The fraction of NK cells recruited to divide at various times was determined from the ratio of the number of triggered receptor complexes (*C_s_* • *t*) to the maximum triggered complexes, without consideration for the threshold number of complexes. The model predictions (solid lines) were compared with results generated from independent experiments (filled circles) where IL-15 concentrations were 9 ng/ml (**B**, n = 3), 25 ng/ml (**C**, n = 4), 50 ng/ml (**D**, n = 2), and 75 ng/ml (**E**, n = 3). The quality of prediction was represented by the normalized root mean squared deviation (NRMSD). The NRMSD of model prediction vs. experimental data were shown as percentages in the upper left of each graph.
(EPS)

Figure S4
**The quantitative influence of high affinity binding model parameters on the steady state cell surface receptor and complex numbers.** Model simulations were performed with the value of the parameter of interest varied by a factor of 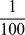, 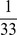, 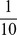, 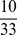, 1, 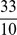, 10, 33, and 100, while the values of all other parameters were held constant. Changes in cell surface receptor and complex numbers as a result of variations in parameter values were shown. Receptor and complex numbers corresponding to different values of the parameter of interest were shown by the dashed curves (with the arrow representing increasing values of the parameter) while the solid curves represented the parameters at their original values. Simulations were performed for model parameters *k_endo_* (**A**), *k_int_* (**B**), *k_s_* (**C**), and *k_syn_′* (**D**), at an IL-15 concentration of 25 ng/ml.(EPS)

Figure S5 **NK cell proliferation response to stimulation from a spectrum of IL-15 concentrations.** **A**. NK cell dose response to IL-15 stimulation (3.9, 7.8, 15.6, 31.3, 62.5, 125, 250, 500, 1000, 2000 ng/ml)were shown as fraction of the maximal thymidine incorporation. Data shown represented the average of five independent experiments. **B**. High affinity receptor binding model simulation of the total number of surface complexes at various IL-15 concentrations (3.9, 9, 25, 50, 75, 125, 250, 500, 1000, 2000 ng/ml). **C**. Population mean division rate was calculated from NK cell experiments with IL-15 concentrations at 3 ng/ml (n = 3), 5 ng/ml (n = 2), 9 ng/ml (n = 3), 25 ng/ml (n = 3), 50 ng/ml (n = 10), 75 ng/ml (n = 3), 100 ng/ml (n = 5), and 200 ng/ml (n = 2).
(EPS)

Figure S6 **Gaussian distribution of the CFSE intensities of dividing NK cells.** The CFSE intensity profiles of dividing NK cells stimulated by 9, 25, and 75 ng/ml of IL-15 were shown for (**A**) 48 hours, (**B**) 61 hours, and (**C**) 78 hours of stimulation. Undivided NK cells were excluded, and the CFSE profiles (black curves) represented the sum of different dividing cohorts. Each CFSE profile was fit with a Gaussian curve (red curves) using OriginPro 7.5 software, and the R^2^ value was shown in each panel. These results were representative of three to four independent experiments.
(EPS)
